# Factors associated with patency of self-expandable metal stents in malignant biliary obstruction

**DOI:** 10.1186/s12876-023-03028-3

**Published:** 2023-11-14

**Authors:** Nottawan Suksai, Patarapong Kamalaporn, Supphamat Chirnaksorn, Sukanya Siriyotha

**Affiliations:** 1 Division of Gastroenterology and Hepatology, Department of medicine, Faculty of Medicine Ramathibodi Hospital, Mahidol University, Bangkok, Thailand; 2 Department of Clinical Epidemiology and Biostatistics, Faculty of Medicine Ramathibodi Hospital, Mahidol University, Bangkok, Thailand

**Keywords:** Self-expandable metal stents, Malignant biliary obstruction

## Abstract

**Introduction:**

Endoscopic self-expandable metal stent (SEMS) placement is the key endoscopic treatment for unresectable malignant biliary obstruction. The benefit of covered SEMS over uncovered SEMS remains unknown as are risk factors for SEMS dysfunction. This study aimed to determine the factors associated with patency of SEMS.

**Methods:**

Patients with unresectable malignant biliary obstruction who underwent endoscopic SEMS placement at Ramathibodi Hospital, during January 2012 to March 2021 were included. Patient characteristics, clinical outcomes and patency of SEMS were collected. The primary outcome were stent patency and factors associated with patency of SEMS. The factors were analyzed by univariate and multivariate analyses. Median days of stent patency, median time of patient survival, rate of reintervention and complications after SEMS placement were collected.

**Results:**

One hundred and fourteen patients were included. SEMS dysfunction was found in 37 patients (32.5%). Size of cancer (Hazard ratio (HR), 1.20, (95% CI 1.02, 1.40), *p* 0.025), presence of stones or sludge during SEMS placement (Hazard ratio (HR), 3.91, (95% CI 1.74, 8.75), *p* 0.001), length of SEMS, 8 cm (HR 2.96, (95% CI 1.06, 8.3), *p* 0.039), and total bilirubin level above 2 mg/dL at one month after SEMS placement (HR 1.14, (95% CI 1.06, 1.22), *p* < 0.001) were associated with SEMS dysfunction. The median stent patency was 97 days. The median patient survival was 133 days, (95% CI 75–165). The rate of reintervention was 86% in patients with SEMS dysfunction.

**Conclusion:**

The size of cancer, presence of stones or sludge during SEMS placement, the length of SEMS, and total bilirubin level above 2 mg/dL at 1 month after SEMS placement were associated with SEMS dysfunction. The median time of stent patency were not statistically different in each type of stent, covered stent, partially covered stent and uncovered stent. Median survival time of patients did not associate with SEMS patency or dysfunction.

## Introduction

Biliary obstruction is a common condition in various types of cancer, including pancreatic cancer, cholangiocarcinoma, duodenal cancer, hepatocellular carcinoma, metastatic cancer, and lymphoma [1]. Most patients with malignant biliary obstruction have a poor quality of life; cholestasis, cholangitis and itching. In the setting of unresectable malignant biliary obstruction, biliary drainage is a recommended palliative treatment for the relief of cholestatic jaundice [2].

Endoscopic biliary drainage is preferred over percutaneous transhepatic drainage because of lower complications, lower risk of malignant peritoneal seeding and shorter length of hospital stay [3]. Self-expandable metal stents (SEMS) have better stent patency than plastic stents, and have been used worldwide for malignant biliary obstruction [2, 4–9].

Partially-covered (PC-SEMS) and fully-covered (FC-SEMS) self-expandable metal stents coated with chemicals [10] were designed to prevent tumor ingrowth which often caused problems in uncovered stent (UC-SEMS). They are removable, so they have a higher risk of stent migration after deployment [11]. Previous meta-analyses showed FC-SEMS and UC-SEMS had different types of adverse events but they did not show significant difference in survival, adverse event rate and stent patency [12–17].

According to prior retrospective trials, concomitant duodenal stent insertion with biliary SEMS was related to lower stent patency in various types of cancer [18] and duodenal invasion itself decreased SEMS patency in pancreatic cancer [19]. PC-SEMS with a proximal uncovered flared end and chemotherapy seemed to improve the patency of the stent [20, 21]. For medication, aspirin (81 mg or more) lowered risk of SEMS occlusion by 51% in the large retrospective study [22]. However, there is no consensus about risk factors for SEMS dysfunction.

The aim of this study is to identify factors associated with the SEMS patency in unresectable malignant biliary obstruction.

## Materials and methods

### Patients

Patients with malignant biliary obstruction who underwent endoscopic SEMS placement at Ramathibodi Hospital, Mahidol University during January 2012 to March 2021 were recruited retrospectively. The data were reviewed from the date of stent placement until November 11th, 2021, the end of follow-up time for this study. The inclusion criteria were: age above 18 years, no previous endoscopic placement of SEMS, presence of unresectable malignant biliary obstruction (advanced disease, metastasis, poor medical conditions) and technically successful placement of UC-SEMS, PC-SEMS, or FC-SEMS. Patients who underwent duodenal stent insertion, or were diagnosed with benign, or uncertain causes of biliary obstruction, or hilar involvement, or lymphoma were excluded. All patients had cytological or histological diagnosis before or on the day of stent placement. Patient characteristics, tumor characteristics, biliary interventions and clinical outcomes were analyzed. The procedures were performed in accordance with relevant guidelines and regulations. Risks and benefits of procedures were discussed. Informed consent was obtained from all subjects and/or their legal guardians. This study was approved by the ethics committee, Ramathibodi Hospital, Mahidol University.

### Procedure

All endoscopic retrograde cholangiopancreatography (ERCP) procedures were performed at the endoscopic unit, Ramathibodi Hospital. All biliary metal stents were made by Boston Scientific, Natick, MA, USA. The type of stent including UC-SEMS, PC-SEMS, FC-SEMS with the diameter 1 cm and the length (6 cm, 8 cm, 10 cm, or 12 cm) of the stent were chosen by the endoscopist preference after evaluating the biliary stricture of each patient. All patients underwent single SEMS placement across the papilla. The position of cystic duct insertion did not determine the length of stent. The PC-SEMS were frequently chosen over the other types, due to concerning of tumor ingrowth, stent migration and reintervention. Normally, no biliary sphincterotomy was performed before stent placement. Antibiotics (ceftriaxone or ciprofloxacin) were given in some patients with possible cholangitis. Bile duct stone or sludge removals were done after stent placement.

### Outcomes and definitions

The primary outcome were SEMS patency and factors associated with SEMS patency.

The secondary outcomes included the median duration of stent patency, median time of patient survival, the rate of reintervention, and complications after SEMS placement. According to TOKYO criteria 2014, stent patency was defined as the time interval between initial stent placement and first time of stent dysfunction. In patient who had no stent dysfunction, stent patency was defined as the time interval between the initial stent placement and death or the end of the study. Stent dysfunction is the composite endpoint of stent obstruction, stent migration, or stent-related events such as cholangitis, pancreatitis, and liver abscess. For transpapillary biliary stenting, technical success was defined as successful deployment of a SEMS in the intended location with sufficient coverage of the stricture [23]. The stent dysfunction was recognized by regular follow-up every 4–6 weeks or visit at Emergency department. Survival time was defined from the time of stent insertion to death or last follow-up.

### Statistical analysis

Sample size was calculated to be at least 89 patients, using the covered-SEMS dysfunction rate of 36.6% according to a previous study [11], 0.10 tolerated error, alpha 0.05 and power 0.8. Patient characteristics were described using mean or median for continuous data, frequency and percentage for categorical data. These data were then compared across clinical outcomes using Chi-square or exact test as appropriate. The statistical evaluation cutoff point using Youden index was applied and identify sensitivity, specificity and ROC with 95% confidence interval. The median time of stent patency was estimated by the Kaplan–Meier method. A multiple cox proportional hazards model was applied to simultaneously SEMS patency on variables whose *p*-value in univariate analysis were less than 0.1. Likelihood ration test was applied to select and keep only significant variables in the final equation. Adjusted hazard ratios (HR) along with 95% confidence interval (CI) were then estimated. All analyses were performed using STATA 17.0. *P* value of less than 0.05 was considered as statistical significance.

## Results

### Patient characteristics

**Fig. 1:**
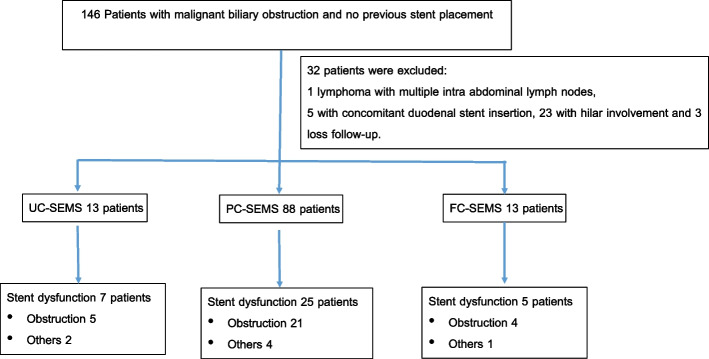
Flow chart of the study

**Fig. 2:**
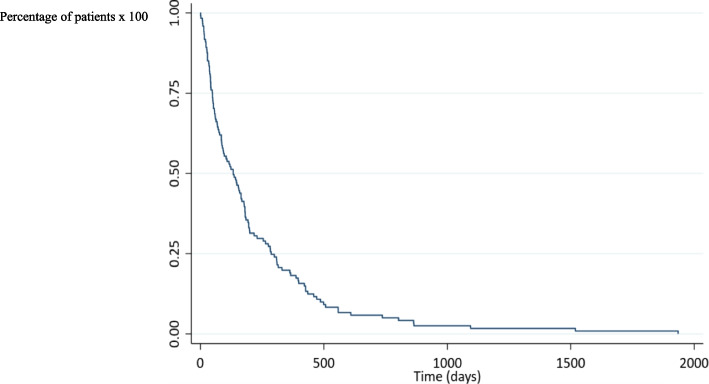
Survival time of all patients

**Fig. 3:**
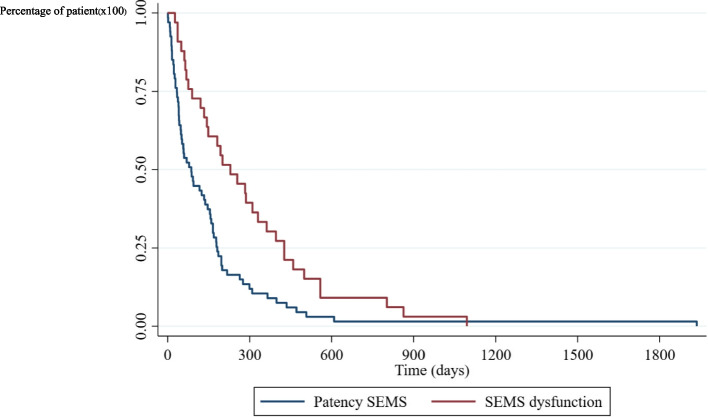
Survival time of patients with and without stent dysfunction

One hundred and forty-six patients who underwent first-time endoscopic biliary SEMS placement for malignant obstruction at our endoscopic unit were identified. Thirty two patients were excluded from this study, one with lymphoma, five with concomitant duodenal stent insertion, twenty three patients with hilar involvement and three with no data on post-procedural follow-up. Nine patients underwent ERCP with plastic stent placement and four patients were sent for percutaneous biliary drainage in this period of time. Finally, 114 patients were included. The patient characteristics are summarized in Table 1. Primary cancers were pancreatic cancer, cholangiocarcinoma, and others, in 67 patients (58.8%), 10 patients (8.8%), and 37 patients (32.5%) respectively. Thirteen patients (11.4%) received UC-SEMS, eighty-eight patients (77.2%) received PC-SEMS, and thirteen patients (11.4%) received FC-SEMS (Table 2; Fig. 1). Two patients developed mild pancreatitis after stent placement and recovered well with supportive treatment. No cholecystitis was detected in this study. The overall median survival time was 133 days, (95% confidence interval 75 to 165), (Fig. 2). The median survival time of patients who experienced stent dysfunction was 229 days, (95% confidence interval 133 to 330) but in patients whose stents were patent until death, the median survival time was 86 days, (95% confidence interval 48 to 146), (Fig. 3). No difference in the median survival time in the uncovered, partially covered and covered stent groups were found.

**Table 1:** Baseline characteristics of patients

Characteristics	*N* = 114
Male, n (%)	53 (46.5)
Age, year, mean (SD)	67.6 (12.4)
Comorbidities	
Metabolic disease, n (%)	58 (50.9)
Cardiac disease, n (%)	39 (34.2)
Liver disease, n (%)	10 (8.8)
Other, n (%)	34 (29.8)
Antiplatelets use, n (%)	9 (7.9)
Ursodeoxycholic acid use, n (%)	20 (17.5)
Perioperative bacterial infection or colonization^a^, n (%)	34 (29.8)
With gallbladder, n (%)	100 (87.7)
Presence of gallstones, n (%)	38 (33.3)
Primary cancer	
Pancreatic cancer, n (%)	67 (58.8)
Cholangiocarcinoma, n (%)	10 (8.8)
Other, n (%)	37 (32.5)
Stage, n (%)	
I/II/III	21 (18.4)
IV	93 (81.6)
Primary tumor size, cm, median (IQR)	3.9 (0.8, 13.2)
Length of stricture, cm, mean (SD)	3.9 (1.4)
Part of obstruction	
Proximal common bile duct, n (%)	9 (7.9)
Mid common bile duct, n (%)	35 (30.7)
Distal common bile duct, n (%)	94 (82.5)
More than one level of common bile duct obstruction, n (%)	27 (23.7)

^a^Positive bacterial culture from bile fluid without evidence of bacterial septicemia

**Table 2:** Procedures and laboratory results

Procedures and laboratory results	Value
Stent type	
UC-SEMS, n (%)	13 (11.4)
PC-SEMS, n (%)	88 (77.2)
FC-SEMS, n (%)	13 (11.4)
Length of stent	
6 cm, n (%)	96 (84.2)
8 cm, n (%)	15 (13.2)
10 cm, n (%)	2 (1.8)
12 cm, n (%)	1 (0.9)
Stent insertion with difficulty, n (%)	10 (8.8)
Percentage of diameter of stent after placement, compared with maximum diameter, median (IQR)	51.1 (0.0, 79.9)
Presence of stones or sludge during SEMS placement, n (%)	23 (20.2)
Total bilirubin before SEMS placement, mg/dL, median (IQR)	8.3 (0.5, 32.5)
Total bilirubin within 72 h after SEMS placement, mg/dL, median (IQR)	3.9 (0.2, 24.2)
Total bilirubin at 1 month after SEMS placement, mg/dL, median (IQR)	1.6 (0.2, 29.3)
Change of total bilirubin: within 72 h, mg/dL, median (IQR)	-3.4 (-17.7, 3.1)
Change of total bilirubin at 1 month, mg/dL, median (IQR)	-6.0 (-31.4, 20.9)
Concurrent tumor treatment, n (%)	72 (63.2)

*UC-SEMS* Uncovered self-expandable metal stent, *PC-SEMS* Partially-covered self-expandable metal stent, *FC-SEMS* Fully-covered self-expandable metal stent

### Biliary SEMS dysfunction and associated risk factors

**Fig. 4:**
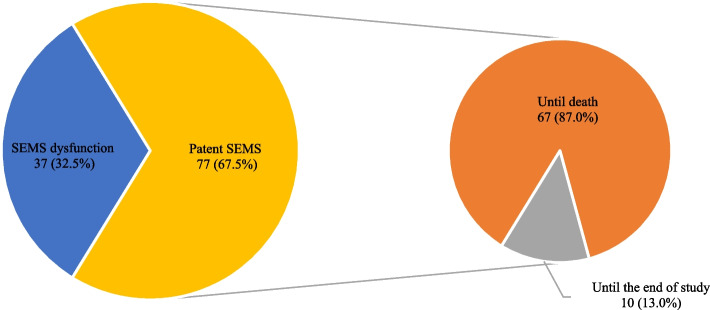
Patency of SEMS in population

SEMS dysfunction was found in 37 patients (32.5%) (Fig. 4). The median time to dysfunction by the Kaplan–Meier method was 97 days. The median time of stent patency were 133, 92, and 147 days in UC-SEMS, PC-SEMS, and FC-SEMS respectively (*p* = 0.309). Comorbidity with size of cancer, presence of stones or sludge during SEMS placement, total bilirubin at 1 month after SEMS placement, length of stent, liver disease, distal common bile duct obstruction, stent insertion with difficulty were potential factors associated with biliary SEMS dysfunction, with *p* < 0.1 in the univariate (Table 3). These factors were subsequently analyzed in the multivariate Cox model. The size of cancer, presence of stones or sludge during SEMS placement, the length of SEMS 8 cm compared to the length of SEMS 6 cm, and total bilirubin level at 1 month after SEMS placement were associated with SEMS dysfunction (Table 3). The total bilirubin levels above 2 mg/dL at 1 month after stent insertion, appeared to be associated with stent dysfunction despite having low sensitivity (43.2%; CI: 27.1–60.5%), specificity (59.7%; CI: 47.9–70.8%) and ROC (51.5%; CI: 41.7–61.3%). The SEMS-related complications which occurred in 37 patients (32.5%), included stent obstruction (30 patients, 81.1%), cholangitis (22 patients, 59.5%), stent migration (5 patients, 13.5%), and pancreatitis (2 patients, 5.4%). Some patients had more than one complication (e.g. stent obstruction and cholangitis occurred in 15 patients).

**Table 3:** Factors association with SEMS patency: A univariate and multivariate analyses by cox proportional hazard model

Factors	Patent SEMS	SEMS dysfunction	Univariate		Multivariate	
	*n* = 77	*n* = 37	HR(95% CI)	*P*-value	HR (95% CI)	*P*-value
Antiplatelets use	5	4	1.53 (0.54, 4.34)	0.426		
Ursodeoxycholic acid use	12	8	1.23 (0.56, 2.71)	0.602		
Primary cancer						
Cholangiocarcinoma	5	5	2.2 (0.8, 5.9)	0.117		
Other	26	11	0.8 (0.4, 1.6)	0.485		
Pancreatic cancer	46	21	1			
Size of cancer, median (IQR)	3.8 (3.0, 4.8)	4.0 (2.8, 5.1)	1.3 (1.1, 1.5)	0.006	1.20 (1.02, 1.40)	0.025
Length of stricture, median (IQR)	4.0 (3.0, 5.0)	4.0 (2.5, 4.0)	0.9 (0.7, 1.1)	0.231		
Current treatment (chemo/ RT/ surgery)	45	27	1.4 (0.7, 3.1)	0.336		
Presence of stones or sludge during SEMS placement	10	13	2.86 (1.43, 5.72)	0.003	3.91 (1.74, 8.75)	0.001
Total bilirubin at 1 month after SEMS placement, mg/dL, median (IQR)	1.6 (0.2–23)	1.8 (0.4, 29.3)	1.12 (1.05, 1.19)	0.001	1.14 (1.06, 1.22)	< 0.001
Length of stent						
10–12	1	1	1.35 (0.18, 10.02)	0.767	2.10 (0.27, 16.12)	0.476
8	9	6	2.57 (1.05, 6.28)	0.039	2.96 (1.06, 8.30)	0.039
6	67	30	1		1	
Liver disease	5	5	2.49 (0.96, 6.45)	0.060		
Perioperative bacterial infection	21	12	1.43 (0.72, 2.88)	0.310		
Proximal common bile duct	5	3	1.43 (0.43, 4.67)	0.559		
Distal common bile duct	66	27	0.49 (0.24, 1.02)	0.056		
More than one level of common bile duct obstruction	25	9	1.29 (0.61, 2.74)	0.506		
Stent type						
FC-SEMS	8	5	0.54 (0.17, 1.71)	0.295		
PC-SEMS	63	25	0.53 (0.23, 1.22)	0.135		
UC-SEMS	6	7	1			
Change of total bilirubin: within 72 h, mg/dL, median (IQR)	-6.1 (-17.7, 3.1)	-2.2 (-15.7, 3.1)	1.04 (0.96, 1.12)	0.315		
Change of total bilirubin at 1 month, mg/dL, median (IQR)	-10.5 (-31.4, 6.5)	-4.0 (-27.8, 20.9)	1.03 (0.98,1.07)	0.249		
Stent insertion with difficulty	4	6	2.35 (0.98, 5.66)	0.056		

*UC-SEMS* Uncovered self-expandable metal stent, *PC-SEMS* Partially-covered self-expandable metal stent, *FC-SEMS* Fully-covered self-expandable metal stent, *CBD* Common bile duct, *VS* Versus, *HR* Hazard ratio

Biliary stones or sludge, tumor ingrowth and food particles were the common causes of SEMS obstruction. According to stent type, obstruction by stones and food particles were mainly seen in PC-SEMS and FC-SEMS. Causes of obstruction and time to stent obstruction for each type of SEMS were showed in Table 4. 32 of 37 patients who had SEMS-related complications required reintervention, i.e., ERCP with common bile duct stones or common bile duct sludge removal (27 patients) and ERCP with stent exchange (5 patients). The reintervention was not done in 5 patients due to terminal stage of disease.

**Table 4:** Time to stent obstruction by various causes; median (range)

	UC-SEMS		PC-SEMS		FC-SEMS	
	n	days	n	days	n	days
Stone/ sludge	1	85	13	97 (12–643)	2	170.5 (147–194)
Food particles	3	157 (133–169)	4	32 (12–245)	1	93
Tumor ingrowth	1	32	2	97 (97,97)	1	207
Tumor overgrowth			2	31.5 (13–50)		

*UC-SEMS *Uncovered self-expandable metal stent, *PC-SEMS *Partially-covered self-expandable metal stent, *FC-SEMS *Fully-covered self-expandable metal stent

## Discussion

This is a tertiary care center retrospective study of 114 patients who received SEMS insertion for malignant biliary obstruction, including uncovered, partially covered and covered types of SEMS. Biliary obstruction by pancreatic cancer and cholangiocarcinoma were the leading indications for SEMS placement, similar to a previous study in Japan [20]. The results of our study showed that median survival time for patients with patent stents was shorter than patients with stent dysfunction. However, this might be easily explained that the median survival time of patients in this study was affected predominantly by the natural history of the disease and the disease progression rather than stent dysfunction. Patients with aggressive or progressive disease died from the malignancy before their stents had time to be occluded. If they had survived longer, stent dysfunction may have occurred similarly in this group. Conversely, patients who lived long enough suffered from stent dysfunction, and so when the patients were classified according to stent patency or dysfunction, we see that survival is actually longer in the stent dysfunction group. Most of the patients (77.2%) received PC-SEMS rather than UC-SEMS or FC-SEMS due to concerns of tumor ingrowth and stent migration, respectively. Rate of stent dysfunction was 32.5%, comparable to an earlier study [2]. Overall median time of stent patency was 97 days. Although FC-SEMS had longer median duration of patency than PC-SEMS and UC-SEMS respectively, this was not statistically significant.

Our results were similar to previous studies in that no particular type of SEMS demonstrated superior patency over other types. Covered metal stents had lower risk of tumor ingrowth but stones, sludge and food particles were found to be the causes of stent obstruction. Data about maintaining a low-fiber diet intake was also collected, but this was not found to be a protective factor for stent dysfunction. Nevertheless, the accuracy of our dietary-intake data may have been limited.

According to our univariate and multivariate analyses, size of cancer was associated with SEMS dysfunction. Normally, the large tumor has shorter doubling time, so the larger tumor in stent dysfunction group could have a higher rate of disease progression and stent occlusion. The presence of stones or sludge during SEMS placement was the second factor associated with SEMS dysfunction. This finding corresponded with the fact that biliary stones and sludge were the main causes of recurrent stent obstruction. Cholestasis can lead to biliary stone formation, so patients with cholestasis have the potential for stent reocclusion by stones or sludge. The stents with 8 cm was the third factor associated with poorer patency compared to 6 cm stents. There may be two possible explanations for this finding. Firstly, distal biliary lesions which needed only 6-cm-long stent may have had lower rates of complication, perhaps due to the small size of the lesion or the distal location of the tumor. Conversely, more proximal lesions that require longer stents may have been more complex. Secondly, the longer stent, 8 cm, may has had more risk of stent dysfunction from stones or sludge because they had more length for the stones to obstruct, as any obstruction along any part of their length is sufficient for dysfunction. Although this study excluded patients with hilar involvement, the long uncovered metallic stents 10 and 12 cm were placed in some patients for covering common bile duct to intrahepatic duct as endoscopist preference. It is likely that not enough 10 and 12 cm stents were used for the analysis to reach statistical significance. The remaining factor associated with SEMS dysfunction was the level of total bilirubin above 2 mg/dL at 1 month after stent insertion. High levels of total bilirubin in spite of SEMS insertion might indicate that the stents have partially opened, resulting in inadequate biliary drainage. In this study, types of SEMS did not affect the overall patency of stent. But SEMS-related complications were different for each type of stent. The reintervention rate was as high as 86% in patients with stent dysfunction. Although a prior study identified the combined placement of duodenal and biliary SEMS as the risk factor for early stent dysfunction, [19] this condition was excluded due to the concern of difficulty of reintervention. Concomitant chemotherapy was considered to be a protective factor for metal stent dysfunction in a prior study [16] but there was no statistical significance in our study.

There were some limitations of current study. Firstly, the retrospective design of our study meant that our data collection was incomplete. Secondly, stent types and length of stents were chosen by endoscopist preference. The types of metal stent are not equally distributed, so the statistical analysis for the causes of stent dysfunction are limited. Moreover, data from a single tertiary center may not be comparable to that from a multicenter study. Further multicenter prospective studies may reduce these limitations.

## Data Availability

The datasets generated and/or analysed during the current study are not publicly available due   to hospital regulation and ethics committee regulation but are available from the corresponding author on reasonable request.
